# Mapping brain–behavior networks using functional and structural connectome fingerprinting in the HCP dataset

**DOI:** 10.1002/brb3.1647

**Published:** 2020-04-30

**Authors:** Ying‐Chia Lin, Steven H Baete, Xiuyuan Wang, Fernando E Boada

**Affiliations:** ^1^ Center for Advanced Imaging Innovation and Research (CAI2R) NYU School of Medicine New York NY USA; ^2^ Center for Biomedical Imaging Department of Radiology NYU School of Medicine New York NY USA

**Keywords:** brain behavior, brain networks, connectivity, connectome fingerprint, functional connectivity, functional structural connectome, individual difference, neuroplasticity, structure connectivity

## Abstract

**Introduction:**

Connectome analysis of the human brain's structural and functional architecture provides a unique opportunity to understand the organization of the brain's functional architecture. In previous studies, connectome fingerprinting using brain functional connectivity profiles as an individualized trait was able to predict an individual's neurocognitive performance from the Human Connectome Project (HCP) neurocognitive datasets.

**Materials and Methods:**

In the present study, we extend connectome fingerprinting from functional connectivity (FC) to structural connectivity (SC), identifying multiple relationships between behavioral traits and brain connectivity. Higher‐order neurocognitive tasks were found to have a weaker association with structural connectivity than its functional connectivity counterparts.

**Results:**

Neurocognitive tasks with a higher sensory footprint were, however, found to have a stronger association with structural connectivity than their functional connectivity counterparts. Language behavioral measurements had a particularly stronger correlation, especially between performance on the picture language test (Pic Vocab) and both FC (*r* = .28, *p* < .003) and SC (*r* = 0.27, *p* < .00077).

**Conclusions:**

At the neural level, we found that the pattern of structural brain connectivity related to high‐level language performance is consistent with the language white matter regions identified in presurgical mapping. We illustrate how this approach can be used to generalize the connectome fingerprinting framework to structural connectivity and how this can help understand the connections between cognitive behavior and the white matter connectome of the brain.

## Graphical abstract

We demonstrate that combining functional connectivity and structural connectivity improves the prediction of neurocognitive performance on specific language tasks. We use a novel tractography‐based connectome approach on structural connectivity. Our findings suggest that the structural connectome information could lead to a better understanding of the relationship between brain architecture and language function. Our findings suggest tractography‐based connectome is particular improve the predictions indicate that the connectome‐based studies can expand the understanding of the relationship between brain connectivity and behavior

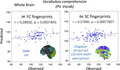

## 1 INTRODUCTION

Recently, a growing number of studies have looked at “connectome fingerprinting” of resting‐state functional magnetic resonance imaging data (Finn et al., 2015;Gratton et al., 2018;Smith et al., 2015;Waller et al., 2017;Yoo et al., 2018). In these studies, a functionally driven parcellation of the brain is used to assess the relationship between the underlying functional connectivity (FC) among the parcels and an individual's performance of various cognitive tasks. They show that this characterization provides a unique fingerprint for each individual that is capable of predicting, a priori, the individual's performance on various cognitive tasks. These studies (Garyfallidis et al., 2018;Jbabdi, Sotiropoulos, Haber, Essen, & Behrens, 2015;Le Bihan & Johansen‐Berg, 2012;Lin et al., 2014;Pestilli, Yeatman, Rokem, Kay, & Wandell, 2014), however, have not established to what extend the underlying brain's neuronal fiber architecture is responsible for the performance of these tasks.

The human connectome project HCP provides an opportunity to investigate the relationship between an individual's underlying brain's neuronal fiber architecture, that is, the individual's structural connectivity (SC) fingerprint, and the individual's brain's ability to perform various cognitive tasks. Previous studies have attempted to investigate the relationship between SC and cognitive performance using SC measures derived from functional parcellations (Hermundstad et al., 2013, 2014;Mišić et al., 2016). These studies found a weaker association between SC and cognitive performance than that between FC and cognitive performance (Fukushima et al., 2018;Zimmermann, Griffiths, Schirner, Ritter, & McIntosh, 2018). This weaker association has been attributed to the lack of extensive neuronal fiber connections between such functional parcels (Chamberland, Bernier, Fortin, Whittingstall, & Descoteaux, 2015;Gomez et al., 2015). To avoid this problem, new “structurally driven” parcellations have been developed that provide a more robust foundation to explore the SC–function relationships of the brain (Cammoun et al., 2012;Daducci et al., 2012;Desikan et al., 2006;Maier‐Hein et al., 2017;Tzourio‐Mazoyer et al., 2002).

The aim of this study was to develop a systematic assessment of brain SC connectivity to identify white matter pathways that can be a significant determinant of behavioral performance. To this end, we calculated a structural connectivity matrix for each individual based on the aforementioned structural parcellations. These structural connectivity matrices are then used to develop a predictive model between specific structural connectivity measures and cognitive performance, such as language comprehension and decoding, working memory, executive function, visual‐spatial processing, and fluid intelligence, using HCP data. Our work demonstrates that such “structural parcellations” can be used to clearly document a strong association between an individual's SC fingerprint and his/her performance of lower‐level cognitive tasks (e.g., reading comprehension). Conversely, this SC fingerprint was also found to have a weaker association with the individual's performance of high‐level cognitive tasks (e.g., working memory).

## 2 MATERIALS AND METHODS

### 2.1 In vivo acquisition

In vivo subject's datasets were downloaded from the Human Connectome Project (HCP) consortium (Van Essen et al., 2012, 2013) led by Washington University, University of Minnesota, and Oxford University. We follow the selection criteria from previous HCP studies (Smith et al., 2015) and include subjects with right‐handed, with four runs of rs‐fMRI (two sessions (REST1/REST2), with two phase‐encoding (LR/RL)), with good movement (mean square displacement <0.1 mm frame‐to‐frame motion), available with quality control DWI data. Less than <200 subjects had valid measurement (too many missing data >700 subjects). After selection of participants from the original HCP sample (*N* = 900) to achieve a final sample size (*N* = 144), we focused on 144 subjects from the December 2015 release (S900, 64/80 male/female, 28.5 ± 4.0 y/o) for which both diffusion MRI and resting‐state fMRI scans are available (Siemens Connectome Skyra 3T, 32‐channel head coil). Resting‐state fMRI time series data (HCP filenames: rfMRI_REST1 and rfMRI_REST2) were acquired with TR/TE = 720/33.1 ms, 2 mm^3^ isotropic resolution, multiband acceleration factor of 8 (Setsompop et al., 2012), and included four runs (two sessions, phase‐encoding (LR/RL), scan time of 14:33 min/run). Diffusion MRI scan parameters were 6 b0‐images, 270 diffusion weighting directions, b‐max = 3,000 s/mm^2^, TR/TE = 5520/89.5 ms, 1.25 mm^3^ isotropic resolution, multiband acceleration factor of 3 (Setsompop et al., 2012), and included six runs (three different gradient table, two phase‐encoding directions (right‐to‐left and left‐to‐right), scan time of 9:50 min/run). Structural imaging (MPRAGE; TR/TE = 2400/2.14 ms, 192 slices, 0.7 mm^3^ isotropic resolution, TI = 1000 ms, parallel imaging (2×, GRAPPA), and total scan time of 7:40 min) was used for registration.

### 2.2 Data preprocessing

The preprocessing of the HCP dataset was performed by the Human Connectome Project consortium as described in Glasser et al. (2013). The processing pipeline included artifact removal, motion correction, and registration to the standard MNI and individual space. For the HCP resting‐fMRI datasets, postprocessing included regressing out the global mean time course of WM and CSF, linear drift removal, artifact removal, frame‐to‐frame motion correction (a Friston 24‐parameter motion model was applied, this included first derivative regression of the mean time courses of the white matter and CSF as well as the global signal), exclusion of motion‐affected datasets (frame‐to‐frame motion, threshold <0.1 mm), and band‐pass filtering (0.01–0.2 Hz) in the BioImage Suite^1^ (Joshi et al., 2011). Spatial smoothing was performed with a Gaussian kernel with a full‐width half‐maximum (FWHM) of 5–6 mm using *3dBlurToFWHM*in AFNI^2^ to reduce the motion confounds in resting‐state fMRI (Cox, 1996;Scheinost, Papademetris, & Constable, 2014)*.* This large amount of smoothing was justified as many contiguous voxels in a single parcellation are averaged when calculating network connectivity. Mean BOLD was calculated within each parcel to reduce the variance associated with the spatial regularization of the template's parcels.

The HCP multiband diffusion‐weighted images were reconstructed using generalized q‐space imaging as implemented in DSIStudio^3^ (Yeh, Wedeen, & Tseng, 2010). Diffusion MRI partial volume effects are similar to those of BOLD fMRI. Smooth parcel boundaries (instead of binary parcel boundaries) were used to reduce the variance associated with parcel size across individuals and for reducing flooding error due to gyral connectivity bias (Van Essen & Ugurbil, 2012;Van Essen et al., 2012, 2014). All parcellations were registered to the individual's diffusion data using *Elastix* ^4^ (Klein, Staring, Murphy, Viergever, & Pluim, 2010). Image alignment was further enhanced by using b‐spline interpolation (Rueckert et al., 1999). Tractography was performed with an optimized deterministic streamline tracking algorithm (Yeh, Verstynen, Wang, Fernández‐Miranda, & Tseng, 2013) (eliminate few fibers due to tissue contamination, turning angle threshold 45⁰, fiber length between 20 and 500mm, one million tracts).

### 2.3 HCP Cognitive measures

As in previous studies (Smith et al., 2015), we focused on a total of 29 nonimaging measures, namely demographic (age, sex, income, education level, etc.) and cognitive performance (including handedness, working memory, attention control, intelligence score, language score, and visual spatial) from the HCP data dictionary.^5^ Specifically, these 29 measures included demographic data: age of participant in years (*Age_in_Yrs),* handedness of participant *(Handedness*)*,* total household income (*SSAGA_Income),* years of education completed *(SSAGA_Educ*), and Pittsburgh sleep quality total scores (*PSQI_Score*); and behavioral data: NIH Toolbox Picture Sequence Memory Test (*PicSeq_Unadj, PicSeq_AgeAdj*), Penn Word Memory Test (*IWRD_TOT, IWRD_RTC (*Gur et al., 2010 *)),* NIH Toolbox List Sorting Working Memory Test *(ListSort_Unadj, ListSort_AgeAdj*), NIH Toolbox Dimensional Change Card Sort Test for executive function/cognitive flexibility (*CardSort_Unadj, CardSort_AgeAdj (*Zelazo et al., 2014 *)),* Delay Discounting for self‐regulation/impulsivity *(DDisc_AUC_200, DDisc_AUC_40K (*Myerson, Green, & Warusawitharana, 2001 *)*), NIH Toolbox Flanker Inhibitory Control and Attention Test for executive function/inhibition (*Flanker_Unadj, Flanker_AgeAdj*), Fluid Intelligence (*PMAT24_A_CR, PMAT24_A_SI, PMAT24_A_RTCR (*Bilker et al., 2012 *)*), NIH Toolbox Oral Reading Recognition Test (*ReadEng_Unadj, ReadEng_AgeAdj (*Gershon et al., 2014 *)),* NIH Toolbox Picture Vocabulary Test *(PicVocab_Unadj, PicVocab_AgeAdj (*Gershon et al., 2014 *)),* NIH Toolbox Pattern Comparison Processing Speed Test *(ProcSpeed_Unadj, ProcSpeed_AgeAdj (*Carlozzi, Beaumont, Tulsky, & Gershon, 2015 *)*), and Variable Short Penn Line Orientation (*VSPLOT_TC, VSPLOT_CRTE, VSPLOT_OFF (*Gur et al., 2010 *)*).

### 2.4 Parcellations

**Figure 1:**
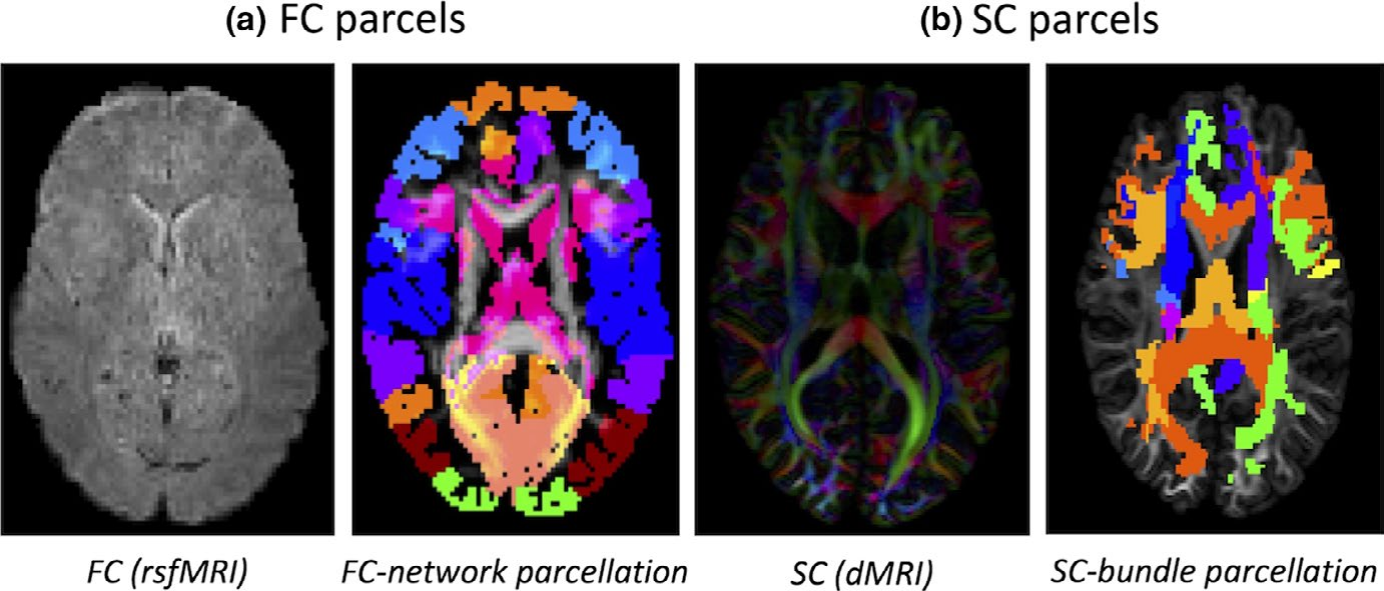
Behavioral traits are correlated with connectivity measures using two methods: FC using FC‐network parcellation (a) and SC using SC‐bundle parcellation (b). These areas are colored according to the cognitive network or majority white matter bundles that they are most connected to. FC‐network parcellation: medial frontal network (MFN), frontal‐parietal network (FPN), default mode network, subcortical network (SUB), somatosensory motor network (SMN), ventral attention network (VAN), visual network (VN), and dorsal attention network (DAN). SC‐bundle parcellation: corpus callosum (CC), cingulum (Cingulum), optic radiation (OR), fornix (Fx)+ posterior (CP)+ anterior commissure (CA), middle + superior+inferior cerebellar peduncle (MCP + SCP+ICP), cortical‐spinal tract + frontal+parietal‐occipital pontine tract (CST + FPT+POPT), and uncinated + superior longitudinal + inferior longitudinal fasciculus (UF + SLF+ILF)

To perform the analysis, we used three different parcellations. The first parcellation employed 268 regions identified on the basis of functional coherence nodes, as recently reported by Shen et al. (Shen, Tokoglu, Papademetris, & Constable, 2013). This parcellation can be subdivided into eight resting‐state cortical and subcortical networks (see Figure 1a) based on their functional activation patterns: (i) medial frontal network (MFN), (ii) frontal‐parietal network (FPN), (iii) default mode network (DMN), (iv) subcortical network (SUB), (v) somatosensory motor network (SMN), (vi) ventral attention network (VAN), (vii) visual network (VN), and (viii) dorsal attention network (DAN) (Finn et al., 2015;Shen et al., 2017). As described before (Finn et al., 2015), the level of activity within these subnetworks is then used to derive each individual's FC fingerprint through Pearson's correlation analysis with the individual's performance data, including Fisher's z‐transform. In this analysis from *r*‐value to *p*‐value, all positive or negative correlations above a *p*‐value threshold (Finn et al., 2015)are considered significant. The correlation analysis includes a leave‐one‐out cross‐validation (LOOCV) training and testing step followed by a false discovery rate (FDR) correction (Benjamini & Hochberg, 1995;Scheinost et al., 2019). No thresholding or binary transformations were applied to the connectivity matrices.

The second parcellation was based on WM bundles as the basis, from which twenty‐five fiber bundle templates were used to develop the structural connectivity matrix, specifically (Figure 1b, (Maier‐Hein et al., 2017)), corpus callosum (CC), cingulum (Cingulum), optic radiation (OR), fornix (Fx)+posterior (CP)+anterior commissure (CA), middle + superior+inferior cerebellar peduncle (MCP + SCP+ICP), cortical‐spinal tract + frontal+parietal‐occipital pontine tract (CST + FPT+POPT), and uncinated + superior longitudinal + inferior longitudinal fasciculus (UF + SLF+ILF). These bundles were further reduced into a set of seven anatomically homologous sets. For each subject, the SC matrices were calculated as either the number of the streamlines connecting each pair of these regions (NS), the normalized length of the connecting streamlines (ML), or the quantitative anisotropy (QA) value (Yeh et al., 2010) along the connecting streamlines. Intersecting bundles providing a physical path between two parcels were also included in the count (Figure 1b).

The final parcellation was based on the AICHA atlas (Joliot et al., 2015), which defines 384 homotopic regions of interest (ROIs) based on their anatomical location. The structural connectivity matrix for this parcellation was calculated as before. The resulting matrix was then subdivided into eight submatrices grouping functionally homologous regions (superior temporal gyrus (STG), superior temporal sulcus (STS), middle temporal gyrus (MTG), superior temporal pole (STP), IFG triangularis (IFGt), IFG orbitalis (IFGo), middle, frontal gyrus (MFG), and angular gyrus (AG) (Del Gaizo, et al., 2017).

### 2.5 HCP individual identification analysis

Individual subjects were identified using functional connectome fingerprints derived from different scanning sessions. To this end, similarity scores SC (*k*), where *k* = 1,…, N, were calculated for each individual between the individual connectivity matrix from the first (reference) session and all connectivity matrices of the second (target) scan sessions (HCP filenames*: rfMRI_REST1* and *rfMRI_REST2*). During the analysis, subject id's and reference and target scans were shuffled, that is, first using DAY1 as the reference then using DAY2 as the reference. The subject was identified as that corresponding to the highest correlation between the reference and target connectome fingerprints. As before, permutation tests (1,000) were performed to increase the statistical significance of the results.

## 3 RESULTS

### 3.1 Individual identification using FC connectome

We first identified the FC connectivity associated with two timepoints to test reproducibility of an individual's identification. As reported elsewhere (Finn et al., 2015;Waller et al., 2017), FC connectivity leads to an identification accuracy between sessions of 90% (Figure S1).

### 3.2 High‐level cognition identification on connectome in FC subnetworks

**Figure 2:**
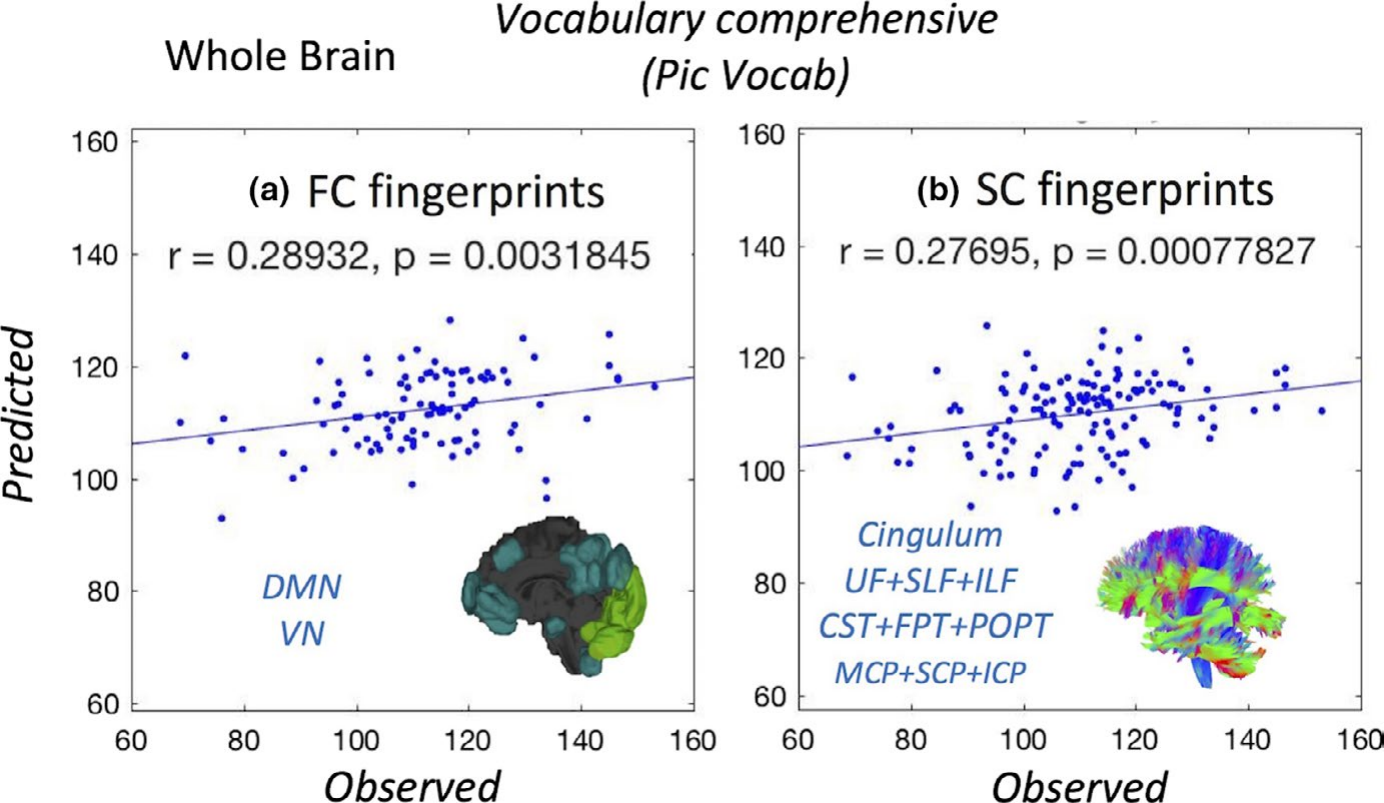
The LOOCV identification of the language test results based on the **negative** correlations of the language test results with functional (a) and structural (b) parcellations

**Figure 3:**
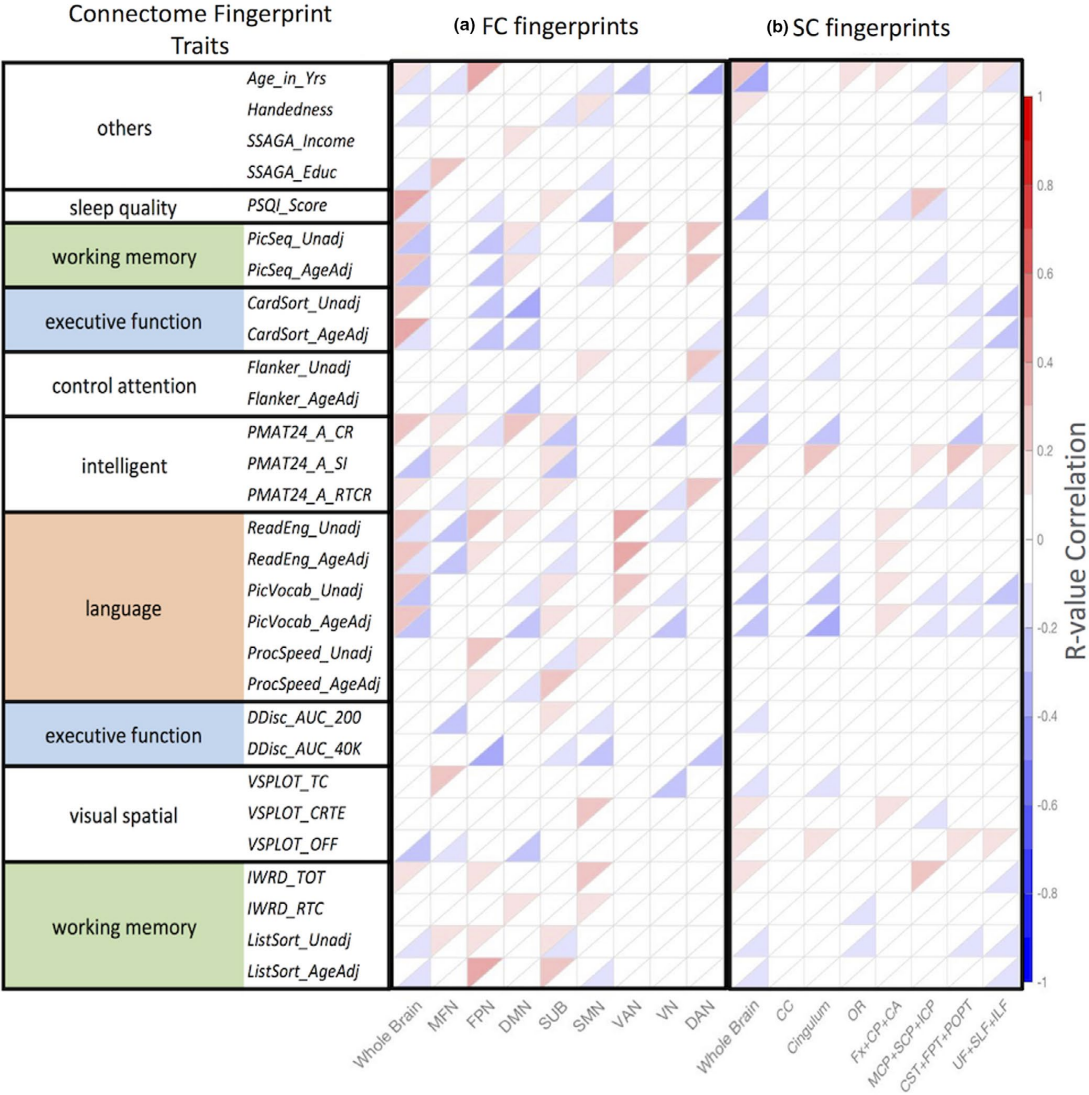
The connectome fingerprints calculated using (a) FC and (b) SC parcellations showing **positive** correlations (in red) and **negative** correlations (in blue) with individual behavior traits in LOOCV model fitting. FC networks and SC bundles (horizontal axis) are related to behavioral traits (vertical axis) and highlight highly significant traits, *p* < .05 with FDR correction (Tables S2‐S3)

We explored the relationship of the FC fingerprints with 29 nonimage behavioral measurements (Table S1). Figure 3a illustrates the positive (upper triangular) and negative (lower triangular) *r*‐values calculated using subject's traits (sleeping quality, working memory, executive function, control attention, intelligent, language, visual spatial) and the aforementioned FC subnetworks (MFN, FPN, DMN, SUB, SMN, VAN, VN, DAN) with a LOOCV method. A similar analysis was also performed using the FC connectivity information without subnetwork partitioning. Significant correlations were found with working memory, language/vocabulary comprehension, and executive function. For the language behavioral measurements, we found a particularly strong correlation between FC and the picture language scores (*Pic Vocab*) (*r* = .28, *p* < .003; Figure 2a). These correlations remain when the same analysis is performed across subnetworks (Figure 3a).

### 3.3 High‐level cognition identification on connectome in SC

For the SC connectome fingerprinting approach in specific white matter bundles, we found strong negative correlations with age, sleep quality, attention control, language, and vocabulary comprehension. These correlations associate white matter bundles and their underlying fiber properties with individual behavioral measurements. For the language behavioral measurements, we found strong negative correlations with language ability in the picture language measurements (*Pic Vocab*) and SC (*r* = .27, *p* < .00077; Figure 2b). Significant positive and negative correlations of the SC subnetworks with behavior traits are summarized in Figure 3b. The results shown identify not only the locations involved in higher cognitive function based on FC, but also using SC (*p* < .01 with FDR correction). Connectome fingerprinting identifies positive and negative correlations of both FC subnetworks and SC of major fiber bundles with behavioral traits. These results indicate the ability of connectome fingerprinting to relate not only FC, but also SC, with neurocognitive measures.

### 3.4 High‐level cognition identification based on SC language networks

**Figure 4:**
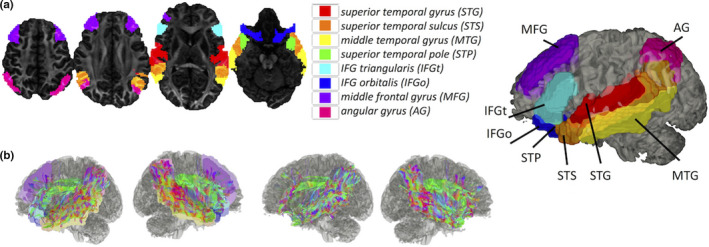
Language‐specific SC ROI used in this study (a), (b) streamlines connecting the language subregions in (a) in a healthy individual. The streamlines are filtered from one million generated streamlines and displayed with (left) and without (right) the subregions. The analysis focuses on eight subnetworks of SC language network. The complete language network, connecting 52 parcels, consists in total of (52x52)/2 connections assigned to 8 subnetworks

**Figure 5:**
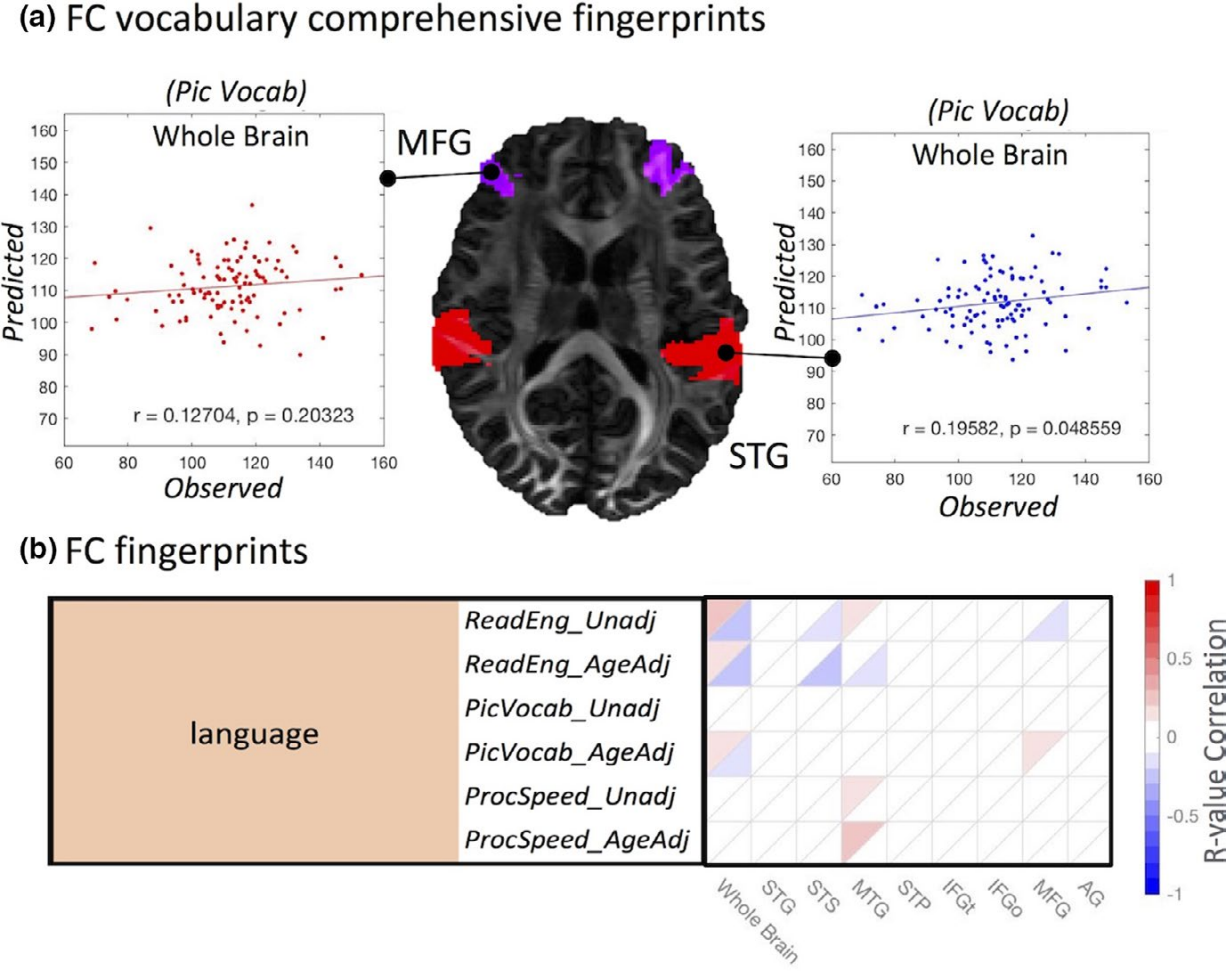
The LOOCV modeling of a language measurement (*Pic Vocab*) calculated using (a) FC based on the **positive** (red) and **negative** (blue) correlations of the language test and related to the most significant language subregions. (b) The connectome fingerprints calculated using FC showing **positive** correlations (in red) and **negative** correlations (in blue) with individual behavior. Language subnetworks (horizontal axis) are related to behavioral traits (vertical axis), and we highlight highly significant traits, *p* < .05 with FDR correction (Table S4). The FC analyses focus on eight language subnetworks (superior temporal gyrus (STG), superior temporal sulcus (STS), middle temporal gyrus (MTG), superior temporal pole (STP), IFG triangularis (IFGt), IFG orbitalis (IFGo), middle, frontal gyrus (MFG), angular gyrus (AG))

**Figure 6:**
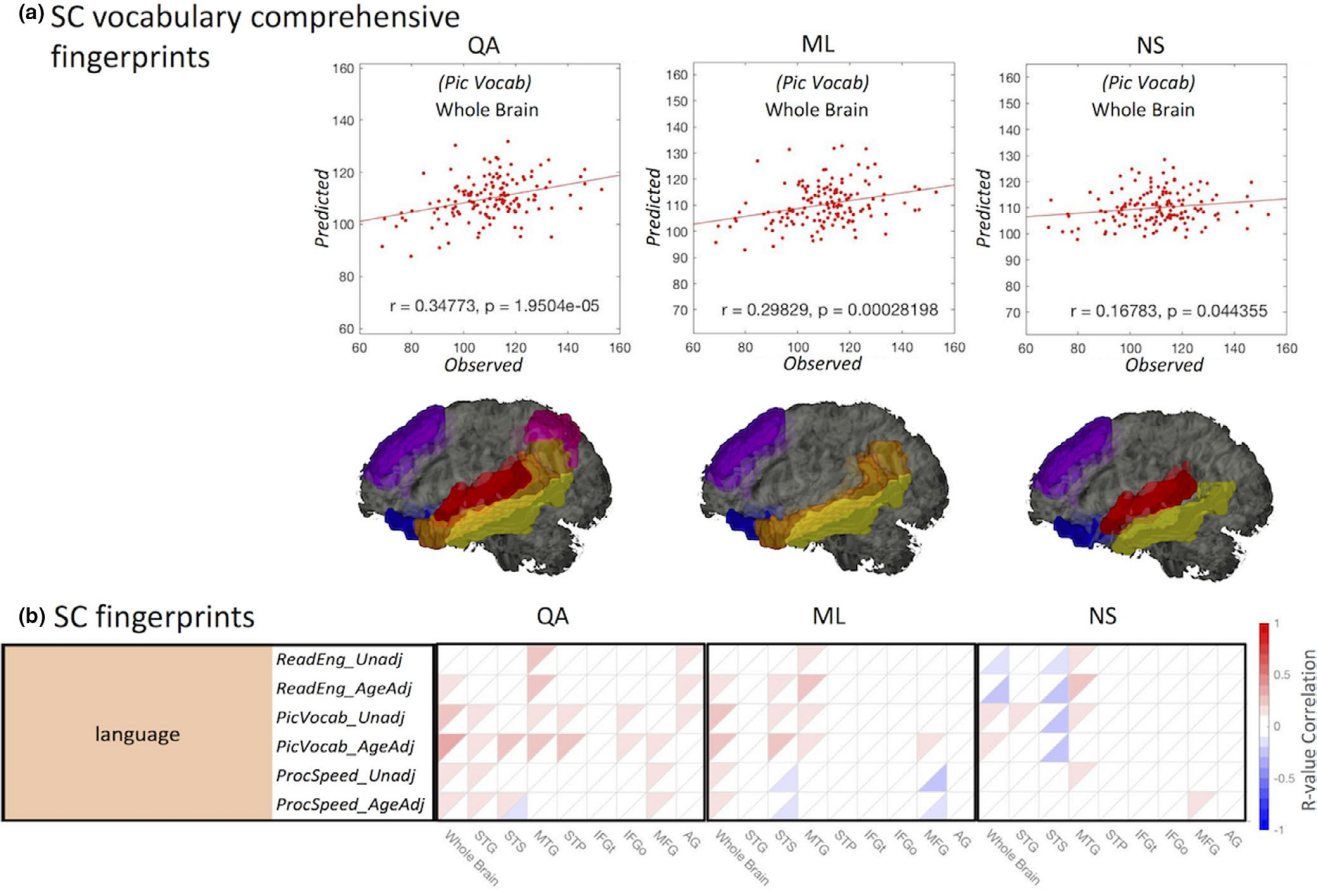
LOOCV modeling of a language measurement (*Pic Vocab*) calculated using (a) SC based on the **positive** correlations of the language test results with QA (quantitative anisotropy), ML (mean streamline length), and NS (normalized number of streamlines). Significant language subnetworks are indicated (lower). (b) The connectome fingerprints calculated using SC showing **positive and negative** correlations with individual behavior traits. Subnetworks (horizontal axis) are related to behavioral traits (vertical axis), and we highlight highly significant traits, *p* < .05 with FDR correction (Tables S5‐S7)

In this section, we focus on SC/FC brain–behavior network based on SC‐derived language subnetworks. In particular, SC and FC connectome fingerprints were derived in eight SC language subnetworks (Figure 4a). Because this study focuses on language‐related fiber bundles (Figure 4b), we further validate these tracts using eight SC language subnetworks (traditional connectome lesion mapping) to relate white matter networks with behavior. The most distinctive language measurements (*Pic Vocab*)correlate negatively (*r* = .19, *p* < .048; Figure 5a) with FC at the individual's whole brain level and at the SC language subnetwork level (*p* < .01 with FDR correction; Figure 5b). Similarly, also SC connectome fingerprints based on quantitative anisotropy (*QA*, *r* = .34, *p* < 1.9504E‐05; Figure 6a), mean fiber length (*ML*, *r* = .29, *p* < .00028; Figure 6a), and normalized number of streamlines (*NS*, *r* = 0.16, *p* < .044; Figure 6a)correlate with language measures both at the individual whole brain level and at the SC language subnetwork level (*p* < .01 with FDR correction; Figure 6b). These eight language networks were the most significant predictors of language function (*Pic Vocab*) when using FC connectome fingerprints. In addition, connectome fingerprinting identifies positive correlations of SC subnetworks with language traits. These results demonstrate that both FC and SC connectome fingerprints can be used to relate the underlying language regions with individual language function.

## 4 DISCUSSION

Previous studies suggest that a systematic view of brain networks could provide a means to describe the functional organization of the brain from its structural anatomy (Hagmann et al., 2008; Honey et al., 2009; Biswal et al., 2010; van den Heuvel & Hulshoff Pol, 2010; Sporns, 2013; Mišić & Sporns, 2016; Griffa et al., 2017; Bennett, Kirby, & Finnerty, 2018; Gratton et al., 2018; Huang et al., 2018).

The functional connectivity (FC), a.k.a. connectome fingerprinting or connectome‐based predictive modeling (CPM (Finn et al., 2015)), framework can be used to derive a quantitative relationship between functional connectivity parameters and cognitive performance. Our results confirm some of these findings and provide a scaffold for the analysis of similar relationships between structural connectivity and cognitive performance. This analysis is, however, inherently more challenging as cognitive performance can be altered on a time scale that is not consistent with changes in structural brain connectivity. Nevertheless, as various cognitive processes are subserved by sensory inputs, their performance is expected to be modulated by the “efficiency’ of the structural connectivity supporting such inputs. Our results support this thesis using three different structural parcellation models. Specifically, we derived fiber bundle structural connectivity parameters based on the major fiber bundles in the brain by (i) using anatomical information to isolate the desired tracts while at the same time eliminating spurious tracts; and (ii) quantifying the number of intersectingss fibers between major fiber bundles. These steps reduce the number of network features that are used to derive subject‐specific correlations (Kahn et al., 2017;Powell, Garcia, Yeh, Vettel, & Verstynen, 2018;Zimmermann et al., 2018) and provide more global measures of structural connectivity than what could be derived from voxel‐wise correlations (Baete et al., 2018; Powell et al., 2018; Yeh, Badre, & Verstynen, 2016).

Using the aforementioned approach, we have established that structural connectivity could be an important determinant of behavioral performance. Specifically, we have shown that subject's performance on vocabulary comprehension tasks has a dependence SC measures that is similar to those shown in previous studies (Bizzi et al., 2012;Del Gaizo, et al., 2017). In terms of language performance, we found a strong correlation between language ability during the picture vocabulary tests and SC fingerprints (*r* = .33). Moreover, our results demonstrate that SC measures have a stronger effect on language performance than their FC counterparts.

In conclusion, our results have identified white matter pathways that have a strong influence on cognitive performance and these structural–functional relationships can be used to infer neurocognitive measures from neuroimaging data.

## CONFLICT OF INTEREST

No conflict of interest has been declared by the author(s).

## AUTHOR CONTRIBUTIONS

S.B. and F.B. conceived of the presented idea. Y.L. and S.B. designed the model and the computational framework and analyzed the data. Y.L., X.W., and S.B. carried out the implementation. Y.L., X.W., and S.B. performed the calculations. Y.L. and S.B. wrote the manuscript with input from all authors. Y.L., S.B., and F.B. conceived the study and were in charge of overall direction and planning.

## Supporting information

Supplementary Material

## Data Availability

Some of the data were provided by the Human Connectome Project (www.humanconnectome.org), WU‐Minn Consortium (Principal Investigators: David Van Essen and Kamil Ugurbil; 1U54MH091657) funded by the 16 NIH Institutes and Centers that support the NIH Blueprint for Neuroscience Research; and by the McDonnell Center for Systems Neuroscience at Washington University. Users must agree to data use terms for the HCP before being allowed access to the data and Connectome DB. Users must also consult with their local IRB or Ethics Committee to use the HCP data. The HCP has implemented a two‐tiered plan for data sharing, with different provisions for handling open access data and Restricted data. Both Open Access data and Restricted data were utilized in the present study. MATLAB scripts were written to perform the analyses described; this code is available from the corresponding author upon reasonable request.

## ENDNOTES

^1^
https://medicine.yale.edu/bioimaging/suite/lands/

^2^
https://afni.nimh.nih.gov/

^3^
http://dsi‐studio.labsolver.org/

^4^
http://elastix.isi.uu.nl/

^5^
https://wiki.humanconnectome.org/display/PublicData/HCP+Data+Dictionary+Public‐+Updated+for+the+1200+Subject+Release
