# Supplementary material for: Mapping brain–behavior networks using functional and structural connectome fingerprinting in the HCP dataset
Source: Brain Behav. 2020 Apr 30;10(6):e01647. doi: 10.1002/brb3.1647 (PMC7303390; doi:10.1002/brb3.1647)
Supplement: Supplementary file 1 — Supplementary Material [file BRB3-10-e01647-s001.docx]

**Supplementary Information**


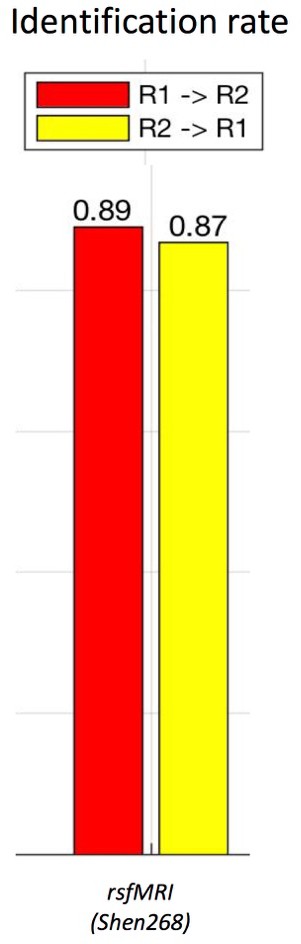


Supplementary Figure S1: FC connectivity at two-time points to test the reproducibility of modeling. Our results reproduce the close to 90% identification of the same subject using FC fingerprints from two separate days. Accuracy for intra-subject identification across session days 1 (R1:REST1) and 2 (R2:REST2).

| Supplementary Table S1. Summary of 29 non-image traits in HCP datasets | | | | | |
| --- | --- | --- | --- | --- | --- |
|  |  | **Sample size N** | **Mean** | **Std. Deviation** | **Skewness (Std. Error)** |
| **Others** | *Age_in_Yrs* | 144 | 28.47 | 4.03 | 0.04 |
|  | *Handedness* | 144 | 67.78 | 39.89 | -2.39 |
|  | *SSAGA_Income* | 143 | 5.09 | 2.12 | -0.28 |
|  | *SSAGA_Educ* | 144 | 15.10 | 1.72 | -0.90 |
| **Sleep quality** | *PSQI_Score* | 144 | 4.77 | 2.95 | 1.62 |
| **Working memory** | *PicSeq_Unadj* | 144 | 112.83 | 13.69 | 0.11 |
|  | *PicSeq_AgeAdj* | 144 | 106.26 | 17.02 | -0.10 |
| **Executive function** | *CardSort_Unadj* | 143 | 114.19 | 10.23 | 0.20 |
|  | *CardSort_AgeAdj* | 143 | 101.35 | 9.83 | -0.19 |
| **Control attention** | *Flanker_Unadj* | 144 | 111.37 | 9.75 | -0.03 |
|  | *Flanker_AgeAdj* | 144 | 101.52 | 9.87 | -0.56 |
| **Intelligent** | *PMAT24_A_CR* | 143 | 17.17 | 4.83 | -0.68 |
|  | *PMAT24_A_SI* | 143 | 2.88 | 3.89 | 1.16 |
|  | *PMAT24_A_RTCR* | 143 | 16295.54 | 8822.80 | 0.80 |
| **Language** | *ReadEng_Unadj* | 144 | 117.30 | 10.39 | 0.16 |
|  | *ReadEng_AgeAdj* | 144 | 107.37 | 14.33 | -0.24 |
|  | *PicVocab_Unadj* | 144 | 117.51 | 9.76 | 0.27 |
|  | *PicVocab_AgeAdj* | 144 | 110.07 | 15.48 | -0.10 |
|  | *ProcSpeed_Unadj* | 144 | 114.23 | 18.08 | -0.05 |
|  | *ProcSpeed_AgeAdj* | 144 | 102.48 | 22.60 | -0.08 |
| **Executive function** | *DDisc_AUC_200* | 139 | 1.97 | 3.02 | 1.56 |
|  | *DDisc_AUC_40K* | 141 | 0.92 | 1.56 | 4.12 |
| **Visual spatial** | *VSPLOT_TC* | 143 | 15.01 | 4.32 | -0.27 |
|  | *VSPLOT_CRTE* | 143 | 1128.46 | 324.68 | 0.90 |
|  | *VSPLOT_OFF* | 143 | 23.77 | 13.94 | 2.50 |
| **Working memory** | *IWRD_TOT* | 143 | 35.79 | 2.93 | -1.17 |
|  | *IWRD_RTC* | 143 | 1524.33 | 217.99 | 0.41 |
|  | *ListSort_Unadj* | 144 | 111.38 | 10.36 | 0.74 |
|  | *ListSort_AgeAdj* | 144 | 103.64 | 11.81 | 0.68 |

.

| **Supplementary Table S2. Summary of the results for 29 non-image traits using the LOOCV modeling analysis in FC connectome fingerprinting** | | | | | | | | | |
| --- | --- | --- | --- | --- | --- | --- | --- | --- | --- |
| **FC fingerprint cognitive function (whole brain)** | **Model response (significant LOOCV highlighted: *p<0.05, FDR-corrected)** |  | **Sample size** | **Significant correlation with behavior (positive)** | **Significant correlation with behavior (negative)** | **Correlation coefficient (positive LOOCV model fit)** | **Correlation coefficient (negative LOOCV model fit)** | **Correlation p-value (positive, FDR corrected)** | **Correlation p-value (negative, FDR corrected)** |
| **Others** | *Age_in_Yrs* |  | 144 | NO | NO | 0.10 | 0.13 | 0.30 | 0.19 |
|  | *Handedness* |  | 144 | NO | NO | 0.00 | 0.16 | 0.84 | 0.11 |
|  | *SSAGA_Income* |  | 143 | NO | NO | 0.00 | 0.00 | 0.28 | 0.14 |
|  | *SSAGA_Educ* | *** | 144 | YES | NO | 0.00 | 0.18 | 0.02 | 0.07 |
| **Sleep quality** | *PSQI_Score* | *** | 144 | YES | NO | 0.32 | 0.19 | 0.00 | 0.06 |
| **Working memory** | *PicSeq_Unadj* | *** | 144 | YES | YES | 0.23 | 0.28 | 0.02 | 0.00 |
|  | *PicSeq_AgeAdj* | *** | 144 | YES | YES | 0.21 | 0.23 | 0.04 | 0.02 |
| **Executive function** | *CardSort_Unadj* | *** | 143 | YES | NO | 0.25 | 0.09 | 0.01 | 0.36 |
|  | *CardSort_AgeAdj* | *** | 143 | YES | NO | 0.31 | 0.16 | 0.00 | 0.10 |
| **Control attention** | *Flanker_Unadj* |  | 144 | NO | NO | 0.00 | 0.00 | 0.41 | 0.28 |
|  | *Flanker_AgeAdj* | *** | 144 | YES | NO | 0.00 | 0.00 | 0.03 | 0.55 |
| **Intelligent** | *PMAT24_A_CR* | *** | 143 | YES | NO | 0.22 | 0.05 | 0.02 | 0.58 |
|  | *PMAT24_A_SI* | *** | 143 | NO | YES | 0.00 | 0.25 | 0.63 | 0.01 |
|  | *PMAT24_A_RTCR* | | 143 | NO | NO | 0.14 | 0.00 | 0.18 | 0.57 |
| **Language** | *ReadEng_Unadj* | *** | 144 | YES | NO | 0.21 | 0.12 | 0.03 | 0.25 |
|  | *ReadEng_AgeAdj* | *** | 144 | YES | NO | 0.27 | 0.13 | 0.01 | 0.20 |
|  | *PicVocab_Unadj* | *** | 144 | YES | YES | 0.27 | 0.22 | 0.01 | 0.03 |
|  | *PicVocab_AgeAdj* | *** | 144 | YES | YES | 0.29 | 0.29 | 0.00 | 0.00 |
|  | *ProcSpeed_Unadj* | | 144 | NO | NO | 0.00 | 0.00 | 0.23 | 0.10 |
|  | *ProcSpeed_AgeAdj* | | 144 | NO | NO | 0.00 | 0.00 | 0.35 | 0.14 |
| **Executive function** | *DDisc_AUC_200* |  | 139 | NO | NO | 0.00 | 0.00 | 0.59 | 0.86 |
|  | *DDisc_AUC_40K* |  | 141 | NO | NO | 0.00 | 0.06 | 0.42 | 0.57 |
| **Visual spatial** | *VSPLOT_TC* |  | 143 | NO | NO | 0.06 | 0.07 | 0.56 | 0.49 |
|  | *VSPLOT_CRTE* |  | 143 | NO | NO | 0.03 | 0.07 | 0.74 | 0.46 |
|  | *VSPLOT_OFF* | *** | 143 | NO | YES | 0.02 | 0.22 | 0.85 | 0.02 |
| **Working memory** | *IWRD_TOT* |  | 143 | NO | NO | 0.18 | 0.05 | 0.07 | 0.65 |
|  | *IWRD_RTC* |  | 143 | NO | NO | 0.00 | 0.00 | 0.09 | 0.94 |
|  | *ListSort_Unadj* |  | 144 | NO | NO | 0.00 | 0.15 | 0.99 | 0.12 |
|  | *ListSort_AgeAdj* |  | 144 | NO | NO | 0.10 | 0.12 | 0.33 | 0.23 |

| **Supplementary Table S3. Summary of the results for 29 non-image traits using the LOOCV modeling analysis in SC connectome fingerprinting** | | | | | | | | | |
| --- | --- | --- | --- | --- | --- | --- | --- | --- | --- |
| **SC fingerprint cognitive function (whole brain)** | **Model response (significant LOOCV highlighted: *p<0.05, FDR-corrected)** |  | **Sample size** | **Significant correlation with behavior (positive)** | **Significant correlation with behavior (negative)** | **Correlation coefficient (positive LOOCV model fit)** | **Correlation coefficient (negative LOOCV model fit)** | **Correlation p-value (positive, FDR corrected)** | **Correlation p-value (negative, FDR corrected)** |
| **Others** | *Age_in_Yrs* | *** | 144 | YES | YES | 0.24 | 0.31 | 0.00 | 0.00 |
|  | *Handedness* |  | 144 | NO | NO | 0.14 | 0.10 | 0.09 | 0.25 |
|  | *SSAGA_Income* |  | 143 | NO | NO | 0.09 | 0.00 | 0.31 | 0.23 |
|  | *SSAGA_Educ* |  | 144 | NO | NO | 0.00 | 0.00 | 0.53 | 0.31 |
| **Sleep quality** | *PSQI_Score* | *** | 144 | NO | YES | 0.07 | 0.22 | 0.38 | 0.01 |
| **Working memory** | *PicSeq_Unadj* |  | 144 | NO | NO | 0.00 | 0.04 | 0.35 | 0.64 |
|  | *PicSeq_AgeAdj* |  | 144 | NO | NO | 0.00 | 0.00 | 0.84 | 0.83 |
| **Executive function** | *CardSort_Unadj* |  | 143 | NO | NO | 0.00 | 0.16 | 0.16 | 0.05 |
|  | *CardSort_AgeAdj* | *** | 143 | YES | NO | 0.00 | 0.08 | 0.01 | 0.37 |
| **Control attention** | *Flanker_Unadj* |  | 144 | NO | NO | 0.00 | 0.16 | 0.99 | 0.05 |
|  | *Flanker_AgeAdj* |  | 144 | NO | NO | 0.00 | 0.11 | 0.20 | 0.18 |
| **Intelligent** | *PMAT24_A_CR* | *** | 143 | NO | YES | 0.00 | 0.23 | 0.46 | 0.01 |
|  | *PMAT24_A_SI* | *** | 143 | YES | YES | 0.26 | 0.00 | 0.00 | 0.00 |
|  | *PMAT24_A_RTCR* | | 143 | NO | NO | 0.00 | 0.07 | 0.62 | 0.39 |
| **Language** | *ReadEng_Unadj* | *** | 144 | NO | YES | 0.00 | 0.18 | 0.06 | 0.03 |
|  | *ReadEng_AgeAdj* | | 144 | NO | NO | 0.00 | 0.15 | 0.16 | 0.07 |
|  | *PicVocab_Unadj* | *** | 144 | NO | YES | 0.00 | 0.28 | 0.16 | 0.00 |
|  | *PicVocab_AgeAdj* | *** | 144 | NO | YES | 0.02 | 0.28 | 0.84 | 0.00 |
|  | *ProcSpeed_Unadj* | | 144 | NO | NO | 0.04 | 0.00 | 0.67 | 0.23 |
|  | *ProcSpeed_AgeAdj* | | 144 | NO | NO | 0.02 | 0.00 | 0.80 | 0.60 |
| **Executive function** | *DDisc_AUC_200* |  | 139 | NO | NO | 0.00 | 0.12 | 0.59 | 0.16 |
|  | *DDisc_AUC_40K* | *** | 141 | YES | NO | 0.00 | 0.00 | 0.00 | 0.35 |
| **Visual spatial** | *VSPLOT_TC* | *** | 143 | YES | NO | 0.00 | 0.14 | 0.00 | 0.10 |
|  | *VSPLOT_CRTE* |  | 143 | NO | NO | 0.13 | 0.06 | 0.13 | 0.45 |
|  | *VSPLOT_OFF* | *** | 143 | YES | NO | 0.20 | 0.00 | 0.02 | 0.07 |
| **Working memory** | *IWRD_TOT* |  | 143 | NO | NO | 0.13 | 0.00 | 0.11 | 0.63 |
|  | *IWRD_RTC* |  | 143 | NO | NO | 0.00 | 0.00 | 0.22 | 0.52 |
|  | *ListSort_Unadj* | *** | 144 | YES | YES | 0.00 | 0.18 | 0.04 | 0.03 |
|  | *ListSort_AgeAdj* |  | 144 | NO | NO | 0.05 | 0.13 | 0.54 | 0.11 |

| **Supplementary Table S4. Summary of the results for 29 non-image traits using the LOOCV modeling analysis in FC connectome fingerprinting (Language networks validation)** | | | | | | | | | |
| --- | --- | --- | --- | --- | --- | --- | --- | --- | --- |
| **SC fingerprint cognitive function (Language networks)** | **Model response (significant LOOCV highlighted: *p<0.05, FDR-corrected)** |  | **Sample size** | **Significant correlation with behavior (positive)** | **Significant correlation with behavior (negative)** | **Correlation coefficient (positive LOOCV model fit)** | **Correlation coefficient (negative LOOCV model fit)** | **Correlation p-value (positive, FDR corrected)** | **Correlation p-value (negative, FDR corrected)** |
| **Others** | *Age_in_Yrs* | *** | 144 | YES | YES | 0.26 | 0.22 | 0.01 | 0.03 |
|  | *Handedness* | *** | 144 | NO | YES | 0.00 | 0.00 | 0.16 | 0.00 |
|  | *SSAGA_Income* | *** | 143 | NO | YES | 0.00 | 0.00 | 0.89 | 0.02 |
|  | *SSAGA_Educ* |  | 144 | NO | NO | 0.18 | 0.10 | 0.07 | 0.32 |
| **Sleep quality** | *PSQI_Score* |  | 144 | NO | NO | 0.02 | 0.00 | 0.84 | 0.16 |
| **Working memory** | *PicSeq_Unadj* | *** | 144 | YES | NO | 0.00 | 0.00 | 0.04 | 0.84 |
|  | *PicSeq_AgeAdj* |  | 144 | NO | NO | 0.04 | 0.06 | 0.69 | 0.53 |
| **Executive function** | *CardSort_Unadj* |  | 143 | NO | NO | 0.00 | 0.12 | 0.13 | 0.23 |
|  | *CardSort_AgeAdj* | *** | 143 | YES | NO | 0.00 | 0.01 | 0.04 | 0.92 |
| **Control attention** | *Flanker_Unadj* |  | 144 | NO | NO | 0.17 | 0.13 | 0.08 | 0.20 |
|  | *Flanker_AgeAdj* |  | 144 | NO | NO | 0.00 | 0.08 | 0.90 | 0.44 |
| **Intelligent** | *PMAT24_A_CR* |  | 143 | NO | NO | 0.17 | 0.09 | 0.09 | 0.36 |
|  | *PMAT24_A_SI* | *** | 143 | YES | YES | 0.20 | 0.25 | 0.05 | 0.01 |
|  | *PMAT24_A_RTCR* | | 143 | NO | NO | 0.03 | 0.00 | 0.73 | 0.23 |
| **Language** | *ReadEng_Unadj* | *** | 144 | YES | YES | 0.26 | 0.26 | 0.01 | 0.01 |
|  | *ReadEng_AgeAdj* | *** | 144 | NO | YES | 0.17 | 0.25 | 0.09 | 0.01 |
|  | *PicVocab_Unadj* |  | 144 | NO | NO | 0.01 | 0.08 | 0.93 | 0.40 |
|  | *PicVocab_AgeAdj* | *** | 144 | NO | YES | 0.13 | 0.20 | 0.20 | 0.05 |
|  | *ProcSpeed_Unadj* | | 144 | NO | NO | 0.06 | 0.00 | 0.54 | 0.23 |
|  | *ProcSpeed_AgeAdj* | | 144 | NO | NO | 0.06 | 0.00 | 0.52 | 0.39 |
| **Executive function** | *DDisc_AUC_200* | *** | 139 | YES | NO | 0.00 | 0.00 | 0.04 | 0.54 |
|  | *DDisc_AUC_40K* | *** | 141 | NO | YES | 0.00 | 0.26 | 0.66 | 0.01 |
| **Visual spatial** | *VSPLOT_TC* |  | 143 | NO | NO | 0.01 | 0.00 | 0.89 | 0.10 |
|  | *VSPLOT_CRTE* | *** | 143 | NO | YES | 0.00 | 0.20 | 0.07 | 0.05 |
|  | *VSPLOT_OFF* |  | 143 | NO | NO | 0.00 | 0.00 | 0.11 | 0.96 |
| **Working memory** | *IWRD_TOT* | *** | 143 | YES | NO | 0.00 | 0.00 | 0.01 | 0.94 |
|  | *IWRD_RTC* | *** | 143 | YES | NO | 0.00 | 0.08 | 0.01 | 0.45 |
|  | *ListSort_Unadj* |  | 144 | NO | NO | 0.10 | 0.00 | 0.31 | 0.76 |
|  | *ListSort_AgeAdj* | *** | 144 | NO | YES | 0.00 | 0.00 | 1.00 | 0.00 |

| **Supplementary Table S5. Summary of the results for 29 non-image traits using the LOOCV modeling analysis in SC connectome fingerprinting (Language networks validation) using QA** | | | | | | | | | |
| --- | --- | --- | --- | --- | --- | --- | --- | --- | --- |
| **SC fingerprint cognitive function (Language areas)** | **Model response (significant LOOCV highlighted: *p<0.05, FDR-corrected)** |  | **Sample size** | **Significant correlation with behavior (positive)** | **Significant correlation with behavior (negative)** | **Correlation coefficient (positive LOOCV model fit)** | **Correlation coefficient (negative LOOCV model fit)** | **Correlation p-value (positive, FDR corrected)** | **Correlation p-value (negative, FDR corrected)** |
| **Others** | *Age_in_Yrs* | *** | 144 | NO | YES | 0.03 | 0.23 | 0.68 | 0.01 |
|  | *Handedness* | *** | 144 | YES | NO | 0.00 | 0.05 | 0.00 | 0.57 |
|  | *SSAGA_Income* |  | 143 | NO | NO | 0.05 | 0.00 | 0.53 | 0.54 |
|  | *SSAGA_Educ* | *** | 144 | YES | NO | 0.00 | 0.00 | 0.00 | 0.97 |
| **Sleep quality** | *PSQI_Score* |  | 144 | NO | NO | 0.00 | 0.04 | 0.11 | 0.62 |
| **Working memory** | *PicSeq_Unadj* |  | 144 | NO | NO | 0.07 | 0.00 | 0.40 | 0.05 |
|  | *PicSeq_AgeAdj* |  | 144 | NO | NO | 0.12 | 0.00 | 0.15 | 0.14 |
| **Executive function** | *CardSort_Unadj* | *** | 143 | YES | NO | 0.22 | 0.00 | 0.01 | 0.69 |
|  | *CardSort_AgeAdj* | | 143 | NO | NO | 0.07 | 0.05 | 0.37 | 0.58 |
| **Control attention** | *Flanker_Unadj* | *** | 144 | YES | NO | 0.21 | 0.02 | 0.01 | 0.85 |
|  | *Flanker_AgeAdj* |  | 144 | NO | NO | 0.04 | 0.00 | 0.66 | 0.14 |
| **Intelligent** | *PMAT24_A_CR* |  | 143 | NO | NO | 0.10 | 0.04 | 0.23 | 0.64 |
|  | *PMAT24_A_SI* |  | 143 | NO | NO | 0.00 | 0.09 | 0.98 | 0.27 |
|  | *PMAT24_A_RTCR* | | 143 | NO | NO | 0.00 | 0.10 | 0.89 | 0.23 |
| **Language** | *ReadEng_Unadj* |  | 144 | NO | NO | 0.01 | 0.00 | 0.90 | 0.30 |
|  | *ReadEng_AgeAdj* | | 144 | NO | NO | 0.16 | 0.00 | 0.06 | 0.27 |
|  | *PicVocab_Unadj* | *** | 144 | YES | NO | 0.27 | 0.02 | 0.00 | 0.85 |
|  | *PicVocab_AgeAdj* | *** | 144 | YES | NO | 0.35 | 0.06 | 0.00 | 0.48 |
|  | *ProcSpeed_Unadj* | *** | 144 | NO | YES | 0.11 | 0.00 | 0.18 | 0.02 |
|  | *ProcSpeed_AgeAdj* | *** | 144 | NO | YES | 0.14 | 0.00 | 0.09 | 0.01 |
| **Executive function** | *DDisc_AUC_200* |  | 139 | NO | NO | 0.00 | 0.00 | 0.96 | 0.05 |
|  | *DDisc_AUC_40K* |  | 141 | NO | NO | 0.13 | 0.00 | 0.13 | 0.81 |
| **Visual spatial** | *VSPLOT_TC* |  | 143 | NO | NO | 0.08 | 0.06 | 0.36 | 0.45 |
|  | *VSPLOT_CRTE* | *** | 143 | YES | NO | 0.00 | 0.02 | 0.01 | 0.81 |
|  | *VSPLOT_OFF* | *** | 143 | NO | YES | 0.04 | 0.23 | 0.67 | 0.01 |
| **Working memory** | *IWRD_TOT* |  | 143 | NO | NO | 0.04 | 0.00 | 0.60 | 0.78 |
|  | *IWRD_RTC* | *** | 143 | NO | YES | 0.00 | 0.00 | 0.15 | 0.00 |
|  | *ListSort_Unadj* |  | 144 | NO | NO | 0.05 | 0.00 | 0.53 | 0.13 |
|  | *ListSort_AgeAdj* | *** | 144 | NO | YES | 0.03 | 0.00 | 0.76 | 0.00 |

| **Supplementary Table S6. Summary of the results for 29 non-image traits using the LOOCV modeling analysis in SC connectome fingerprinting (Language networks validation) using ML** | | | | | | | | | |
| --- | --- | --- | --- | --- | --- | --- | --- | --- | --- |
| **SC fingerprint cognitive function (Language areas)** | **Model response (significant LOOCV highlighted: *p<0.05, FDR-corrected)** |  | **Sample size** | **Significant correlation with behavior (positive)** | **Significant correlation with behavior (negative)** | **Correlation coefficient (positive LOOCV model fit)** | **Correlation coefficient (negative LOOCV model fit)** | **Correlation p-value (positive, FDR corrected)** | **Correlation p-value (negative, FDR corrected)** |
| **Others** | *Age_in_Yrs* | *** | 144 | NO | YES | 0.00 | 0.21 | 0.07 | 0.01 |
|  | *Handedness* | *** | 144 | YES | NO | 0.00 | 0.06 | 0.03 | 0.47 |
|  | *SSAGA_Income* | *** | 143 | NO | YES | 0.00 | 0.00 | 0.50 | 0.04 |
|  | *SSAGA_Educ* |  | 144 | NO | NO | 0.00 | 0.09 | 0.06 | 0.26 |
| **Sleep quality** | *PSQI_Score* |  | 144 | NO | NO | 0.02 | 0.05 | 0.80 | 0.54 |
| **Working memory** | *PicSeq_Unadj* |  | 144 | NO | NO | 0.00 | 0.00 | 0.97 | 0.85 |
|  | *PicSeq_AgeAdj* |  | 144 | NO | NO | 0.00 | 0.00 | 0.56 | 0.81 |
| **Executive function** | *CardSort_Unadj* |  | 143 | NO | NO | 0.07 | 0.00 | 0.43 | 0.14 |
|  | *CardSort_AgeAdj* | | 143 | NO | NO | 0.10 | 0.00 | 0.24 | 0.74 |
| **Control attention** | *Flanker_Unadj* | *** | 144 | YES | NO | 0.19 | 0.00 | 0.02 | 0.24 |
|  | *Flanker_AgeAdj* |  | 144 | NO | NO | 0.16 | 0.02 | 0.06 | 0.86 |
| **Intelligent** | *PMAT24_A_CR* | *** | 143 | NO | YES | 0.08 | 0.00 | 0.32 | 0.02 |
|  | *PMAT24_A_SI* |  | 143 | NO | NO | 0.00 | 0.11 | 0.10 | 0.19 |
|  | *PMAT24_A_RTCR* | | 143 | NO | NO | 0.03 | 0.03 | 0.69 | 0.77 |
| **Language** | *ReadEng_Unadj* | *** | 144 | NO | YES | 0.04 | 0.00 | 0.66 | 0.01 |
|  | *ReadEng_AgeAdj* | *** | 144 | NO | YES | 0.14 | 0.00 | 0.08 | 0.02 |
|  | *PicVocab_Unadj* | *** | 144 | YES | YES | 0.25 | 0.00 | 0.00 | 0.03 |
|  | *PicVocab_AgeAdj* | *** | 144 | YES | NO | 0.30 | 0.00 | 0.00 | 0.38 |
|  | *ProcSpeed_Unadj* | | 144 | NO | NO | 0.15 | 0.00 | 0.06 | 0.10 |
|  | *ProcSpeed_AgeAdj* | | 144 | NO | NO | 0.11 | 0.00 | 0.20 | 0.45 |
| **Executive function** | *DDisc_AUC_200* |  | 139 | NO | NO | 0.04 | 0.00 | 0.65 | 0.15 |
|  | *DDisc_AUC_40K* |  | 141 | NO | NO | 0.06 | 0.11 | 0.51 | 0.20 |
| **Visual spatial** | *VSPLOT_TC* |  | 143 | NO | NO | 0.00 | 0.05 | 0.67 | 0.53 |
|  | *VSPLOT_CRTE* | *** | 143 | YES | NO | 0.00 | 0.04 | 0.00 | 0.65 |
|  | *VSPLOT_OFF* |  | 143 | NO | NO | 0.03 | 0.14 | 0.76 | 0.09 |
| **Working memory** | *IWRD_TOT* |  | 143 | NO | NO | 0.00 | 0.09 | 0.66 | 0.30 |
|  | *IWRD_RTC* |  | 143 | NO | NO | 0.00 | 0.00 | 0.77 | 0.05 |
|  | *ListSort_Unadj* | *** | 144 | YES | NO | 0.00 | 0.00 | 0.00 | 0.98 |
|  | *ListSort_AgeAdj* | *** | 144 | YES | NO | 0.00 | 0.02 | 0.03 | 0.80 |

| **Supplementary Table S7. Summary of the results for 29 non-image traits using the LOOCV modeling analysis in SC connectome fingerprinting (Language networks validation) using NS** | | | | | | | | | |
| --- | --- | --- | --- | --- | --- | --- | --- | --- | --- |
| **SC fingerprint cognitive function (Language areas)** | **Model response (significant LOOCV highlighted: *p<0.05, FDR-corrected)** |  | **Sample size** | **Significant correlation with behavior (positive)** | **Significant correlation with behavior (negative)** | **Correlation coefficient (positive LOOCV model fit)** | **Correlation coefficient (negative LOOCV model fit)** | **Correlation p-value (positive, FDR corrected)** | **Correlation p-value (negative, FDR corrected)** |
| **Others** | *Age_in_Yrs* |  | 144 | NO | NO | 0.00 | 0.09 | 0.31 | 0.26 |
|  | *Handedness* |  | 144 | NO | NO | 0.00 | 0.07 | 0.64 | 0.42 |
|  | *SSAGA_Income* |  | 143 | NO | NO | 0.00 | 0.14 | 0.49 | 0.08 |
|  | *SSAGA_Educ* |  | 144 | NO | NO | 0.00 | 0.00 | 0.99 | 0.57 |
| **Sleep quality** | *PSQI_Score* |  | 144 | NO | NO | 0.00 | 0.00 | 0.07 | 0.10 |
| **Working memory** | *PicSeq_Unadj* |  | 144 | NO | NO | 0.00 | 0.00 | 0.20 | 0.40 |
|  | *PicSeq_AgeAdj* |  | 144 | NO | NO | 0.06 | 0.00 | 0.49 | 0.27 |
| **Executive function** | *CardSort_Unadj* |  | 143 | NO | NO | 0.11 | 0.00 | 0.19 | 0.65 |
|  | *CardSort_AgeAdj* | | 143 | YES | NO | 0.20 | 0.00 | 0.02 | 0.68 |
| **Control attention** | *Flanker_Unadj* |  | 144 | NO | NO | 0.08 | 0.00 | 0.33 | 0.54 |
|  | *Flanker_AgeAdj* |  | 144 | NO | NO | 0.14 | 0.00 | 0.11 | 0.34 |
| **Intelligent** | *PMAT24_A_CR* | *** | 143 | YES | NO | 0.18 | 0.02 | 0.03 | 0.84 |
|  | *PMAT24_A_SI* | *** | 143 | NO | YES | 0.10 | 0.18 | 0.26 | 0.03 |
|  | *PMAT24_A_RTCR* | *** | 143 | NO | YES | 0.13 | 0.00 | 0.11 | 0.03 |
| **Language** | *ReadEng_Unadj* |  | 144 | NO | NO | 0.00 | 0.16 | 0.73 | 0.06 |
|  | *ReadEng_AgeAdj* | *** | 144 | NO | YES | 0.06 | 0.22 | 0.50 | 0.01 |
|  | *PicVocab_Unadj* |  | 144 | NO | NO | 0.12 | 0.00 | 0.17 | 0.97 |
|  | *PicVocab_AgeAdj* | *** | 144 | YES | NO | 0.17 | 0.09 | 0.04 | 0.26 |
|  | *ProcSpeed_Unadj* | | 144 | NO | NO | 0.02 | 0.00 | 0.79 | 0.58 |
|  | *ProcSpeed_AgeAdj* | | 144 | NO | NO | 0.00 | 0.00 | 0.47 | 0.82 |
| **Executive function** | *DDisc_AUC_200* | *** | 139 | NO | YES | 0.15 | 0.00 | 0.07 | 0.00 |
|  | *DDisc_AUC_40K* | *** | 141 | NO | YES | 0.05 | 0.00 | 0.54 | 0.00 |
| **Visual spatial** | *VSPLOT_TC* |  | 143 | NO | NO | 0.09 | 0.04 | 0.30 | 0.66 |
|  | *VSPLOT_CRTE* | *** | 143 | YES | NO | 0.00 | 0.00 | 0.01 | 0.13 |
|  | *VSPLOT_OFF* |  | 143 | NO | NO | 0.07 | 0.15 | 0.40 | 0.07 |
| **Working memory** | *IWRD_TOT* |  | 143 | NO | NO | 0.00 | 0.00 | 0.77 | 0.39 |
|  | *IWRD_RTC* |  | 143 | NO | NO | 0.01 | 0.04 | 0.92 | 0.62 |
|  | *ListSort_Unadj* | *** | 144 | NO | YES | 0.06 | 0.00 | 0.47 | 0.00 |
|  | *ListSort_AgeAdj* |  | 144 | NO | NO | 0.00 | 0.00 | 0.38 | 0.06 |
